# Influence of Nano Silicon and Nano Selenium on Root Characters, Growth, Ion Selectivity, Yield, and Yield Components of Rice (*Oryza sativa* L.) under Salinity Conditions

**DOI:** 10.3390/plants10081657

**Published:** 2021-08-11

**Authors:** Shimaa A. Badawy, Bassiouni A. Zayed, Sherif M. A. Bassiouni, Ayman H. A. Mahdi, Ali Majrashi, Esmat F. Ali, Mahmoud F. Seleiman

**Affiliations:** 1 Agronomy Department, Faculty of Agriculture, Kafrelshiekh University, Kafrelsheikh 33516, Egypt; shamsbadawy2000@yahoo.com; 2 Rice Research and Training Center RRTC, Agriculture Research Center, Field Crops Research Institute, Sakha 33717, Egypt; bas.zayed@gmail.com (B.A.Z.); sherif_maher2002@yahoo.com (S.M.A.B.); 3 Agronomy Department, Faculty of Agriculture, Beni-Suef University, Beni Suef 62521, Egypt; drayman.hamdy@agr.bsu.edu.eg; 4 Department of Biology, College of Science, Taif University, P.O. Box 11099, Taif 21944, Saudi Arabia; aa.majrashi@tu.edu.sa (A.M.); a.esmat@tu.edu.sa (E.F.A.); 5 Plant Production Department, College of Food and Agriculture Sciences, King Saud University, P.O. Box 2460, Riyadh 11451, Saudi Arabia; 6 Department of Crop Sciences, Faculty of Agriculture, Menoufifia University, Shibin El-Kom 32514, Egypt

**Keywords:** nano-silicon, nano-selenium, productivity, ion selectivity, *Oryza sativa* L., salinity

## Abstract

Rice production under salinity stress is a critical challenge facing many countries, particularly those in arid and semi-arid regions. This challenge could be handled by applying novel approaches to overcome yield limiting factors and improve resource use efficiency. The usage of nanoparticles (NPs) could be a beneficial approach to managing the growing problem of soil salinity. The aim of our study was to investigate the advantageous effects of soaking and foliar application of silicon (Si) and selenium (Se), (NPs-Si at 12.5 mg L^−1^ and NPs-Se at 6.25 mg L^−1^) on root characteristics, moropho-physiological traits, and yields of two rice varieties (i.e., Giza 177 as a salt sensitive and Giza 178 as a salt tolerant) grown in saline soil compared to untreated plants (control treatment). Results showed that soaking NPs-Se resulted in the highest value of root thickness for Giza 178 (0.90 mm, 0.95 mm) and root volume (153.30 cm^3^, 154.30 cm^3^), while Giza 177 recorded 0.83 mm, 0.81 mm for root thickness and 143.30 cm^3^, 141.30 cm^3^ for root volume in the 2018 and 2019 seasons, respectively. Soaking NPs-Se, NPs-Si and foliar application of NPs-Se at BT resulted in the highest relative water content and dry matter, while foliar application of NPs-Si at BT gave the highest leaf area index of rice plants compared to the other treatments. Giza 178 (i.e., salt tolerant variety) significantly surpassed Giza 177 (i.e., salt sensitive variety) in the main yield components such as panicle number and filled grains/ panicle, while Giza 177 significantly exceeded Giza 178 in the panicle weight, 1000-grain weight, and unfilled grains number/ panicle. Soaking NPs-Se and foliar application of NPs-Si at BT resulted in the highest grain yield of 5.41 and 5.34 t ha^−1^ during 2018 and 5.00 and 4.91 t ha^−1^ during 2019, respectively. The salt sensitive variety (Giza 177) had the highest Na^+^ leaf content and Na^+^/K^+^ ratio as well as the lowest K+ leaf content during both seasons. Applying nano nutrients such as NPs-Si and NPs-Se improved the yield components of the salt sensitive variety (Giza 177) by enhancing its ion selectivity. Both NPs-Si and NPs-Se had almost the same mode of action to mitigate the harmful salinity and enhance plant growth, and subsequently improved the grain yield. In summary, the application of NPs-Si and NPs-Se is recommended as a result of their positive influence on rice growth and yield as well as minimizing the negative effects of salt stress.

## 1. Introduction

Soil salinization is a critical concern worldwide and has expanded significantly, resulting in crop yield losses [1]. It is a major limitation reducing agricultural productivity on almost 20% of the world’s cultivated and irrigated land [2]. Because of its semi-arid zone weather, sea water, fresh water shortage, and climate change, Egypt has an ongoing salinity problem [3]. Salinity impacts practically every element of plant physiology and biochemistry, resulting in a considerable reduction in yield.

According to the classification of crop tolerance to salinity, the rice crop falls within the sensitive division, ranging from 0 to 8 dS m^−1^ of soil water [4]. Rice susceptibility to salinity stress varies with growth stage. It is very sensitive to the salinity during germination, immature seedling, and early growth phases [5,6]. In contrast, rice is relatively salt tolerant at germination and, in some situations, is unaffected by the high level of salt stress [7]. Seed germination, seedling emergence, and their survival are especially susceptible to the salt substrate [8,9]. The combined effects of high osmotic potential and particular ion toxicity can greatly impede seed germination and soil growth [10]. In general, rice at the young seedling stage is particularly sensitive, which affects the density of the plants in the salt fields [11]. The sensitivity and levels of tolerance for successful crop production in a saline environment of the varieties should be identified during early seeding.

Salinity is a serious stress condition nowadays [12], and is one of the most important environmental concerns influencing crop growth [13]. It is one of the world’s most important environmental challenges in agriculture, along with drought. This issue is especially severe in the world’s arid and semi-arid regions [14]. Salinity not only causes discrepancies between the mean and potential yields, but also causes yield loss from year to year. It directly influences seed germination and plant growth by interacting with metabolic rates and pathways within plants [14]. Among the nutritional elements, silicon (Si) is thought to be one of the most beneficial to plant life [15]. Si’s potential to mitigate the harmful effects of salt stress on plant growth is well documented [16]. Several studies have revealed that silicon improves salt tolerance in a variety of crop species [16,17,18]. The application of micronutrients can promote the growth and yield of crops by increasing plant hydration status, changing the ultrastructure of leaf organelles, activating plant defense mechanisms, and mitigating particular ions [16,19].

A low nitrogen use efficiency can result in an intensive application of conventional fertilizers to increase crop yields. Nevertheless, the intensive application of conventional fertilizers can cause severe environmental problems, for example, groundwater pollution, soil degradation, and water eutrophication as and can also reduce the profit margin of farmers [20,21,22,23,24,25]. Nanoparticles (NPs) have attracted the attention of researchers due to its distinct physicochemical properties when compared to bulk particles [20]. Agricultural experts have recently focused their attention on nano-compound materials [20,21,22]. NPs have distinct properties due to their small size [20]. They can, for example, alter the physicochemical qualities when compared to the bulk materials [20]. NPs have a larger surface area than bulk materials, and as a result, their solubility and surface reactivity are higher [20,23]. Nano-silicon (NPs-Si) is one of the helpful NPs that have been reported to be beneficial in modern agriculture [20,21,24]. Moreover, the mesoporous structure of NPs-Si can make it a proper nanocarrier for different molecules that are considered beneficial in agricultural productivity [20]. However, the knowledge of the mode of action for the NPs on the plant growth and its development remains limited [20]. The application of NPs-Si improved the development and quality of Larix seedlings [25] and improved the productivity of strawberry plants grown under drought stress [21]. In addition, the application of Zn-NPs enhanced the growth, physiological, and yield characteristics of plants grown in a contaminated soil compared with untreated soil [26], while the application of NPs-Si improved the productivity of crops grown under salinity stress [27]. Although there are several references on the interaction of salinity and Si in higher plants, there is currently little information available on the potential benefits of NPs-Si to minimize and mitigate salt stress damage in plants.

Selenium (Se) is regarded as one of the most important micronutrients in human and animal nutrition, but its importance for higher plants remains unproven [28]. Se has numerous applications in food and feed via various biofortification methods [29,30]. It can be found in different varieties of forms including selenite, selenate, nanoparticles of selenium (NPs-Se), and selenoproteins [28]. There are several published investigations on the essential impact of NPs-Se on humans and animals under stressful conditions, but few investigations have been conducted on higher plants [31]. Exogenous foliar application of NPs-Se can enhance plant growth by activating the antioxidant system under nutrient deficiency in the sandy soils [32] as well as improve the crop yield and quality in arid zones [33]. The application of NPs-Se can alleviate the toxicity of Cd and Pb in plants [34,35], mitigate the freezing stress on the plant photosynthesis [36], and improve plant growth such as lettuce and strawberry grown under salt stress [34,35,37]. Plants absorb the soluble nutrient ions in the same way that they absorb dissolved traditional fertilizer ions. However, because of the significantly smaller particle sizes and higher specific surface areas of NPs for different nutrients, their dissolving rate and extent in water/soil solution should be greater than that of equivalent bulk materials [38].

Therefore, the current study aimed to elucidate the potential effect of the application of NPs-Si (12.5 mg L^−1^) and NPs-Se (6.25 mg L^−1^) on the root characteristics, growth, development, ion selectivity, and productivity of rice (*Oryza sativa* L.) at different growth stages under salinity stress conditions compared to untreated plants (control; distilled water). The hypothesis was that the soaking or exogenous application of NPs-Si and NPs-Se at different growth stages can mitigate the salinity stress and improve the productivity of rice grown in saline soil.

## 2. Material and Methods

### 2.1. Treatments, Plant Materials, and Soil Analysis

The current study was carried out at the Experimental Farm of El-Sirw Agricultural Research Station, Damietta Governorate (latitude: 31°24′84″ and longitude: 31°65′34″), Egypt, during 2018 and 2019 seasons to study the response of the root characteristics, moro-physiological, and yield traits of Giza 177 as the salt sensitive variety and Giza 178 as the salt tolerant variety into two nanoparticle (NP) nutrients of silicon (NPs-Si) and selenium (NPs-Se) application over different times and modes. The treatments were the control (distilled water application), grain soaking in NPs-Si, grain soaking in NPs-Se, NPs-Si foliar application at mid tillering stage (MT), NPs-Se foliar application at MT, NPs-Si foliar application at panicle initiation (PI), NPs-Se foliar application at PI, NPs-Si foliar application at mid booting stage (BT), and NPs-Se foliar application at BT. In soaking method (before sowing), grains were soaked in the solution of NPs-Si (12.5 mg/L) and NPs-Se (6.25 mg/L) overnight at room temperature in the dark. Then, the solution was filtered and grains were taken for sowing. Concerning the foliar application, plant leaves were fully treated with NPs-Si (12.5 mg/L) and NPs-Se (6.25 mg/L) at the above-mentioned growth stages.

NPs-Se solution was prepared at the Agricultural Microbiology Department, Soils, Water and Environment Research Institute (SWERI), Sakha Agricultural Research Station, Agriculture Research Center (ARC), Kafr El-Sheikh, Egypt. A biological procedure was used to prepare NPs-Se (50–100 nm) through using *Lactobacillus casei* according to Eszenyi et al. [39]. In this method, sodium selenite (Na_2_SeO_3_) as a source of selenium is converted into NPs-size in a red color by using probiotic yogurt bacteria (*Lactobacillus casei*) in a fermentation procedure using MRS media. After that, the medium was centrifuged at 6000 rpm for 10–15 min, and then shaken in the incubator for 36–48 h at 37 °C. The characteristics of the used NPs-Si (SiO_2_) was 260–320 m^2^ g^−1^ for specific surface area, 4–4.5 for pH, and 10 nm for diameter.

The treatments (two rice varieties × nine treatments of NPs applied either as grain soaking or foliar applications) were arranged in a split plot design with four replications. The rice varieties were distributed in the main plots, while the NP treatments were located in the sub-plots. The experimental soil was saline clay and its chemical analysis properties were analyzed according to Black et al. [40]. The soil analysis is presented in Table 1.

The nursery land was fertilized with calcium super phosphate (15.5% P_2_O_5_) at the rate of 50 kg P_2_O_5_ ha^−1^ before plowing. Nitrogen in the form of urea (46.0% N) was added at the rate of 165 kg N ha^−1^ after the last plowing before leveling and immediately before sowing. Rice grains at the rate of 140 kg ha^−1^ were soaked in fresh water for 24 h and incubated for another 48 h. Thereafter, it was broadcasted with 2–3 cm standing water in the nursery on 25 April, in both seasons.

Weeds were chemically controlled with Saturn 50% [S-(4-Chlorophenol methyl) diethyl carbamothioate] at the rate of 4.8 L ha^−1^. The permanent field was fertilized with 50 kg P_2_O_5_ ha^−1^ before plowing. At 30 days from sowing, seedlings were carefully pulled from the nursery and manually transplanted into plots (10 m^2^) in 20 × 20 cm spacing at the rate of 3 seedlings hill^−1^. Seven days after transplanting, the herbicide Saturn 50% was added at the rate of 4.8 L ha^−1^. Plots were kept flooded until two weeks before harvesting. Potassium fertilizer in the form of potassium sulfate (48% K_2_O) at rate of 60 kg K_2_O ha^−1^ was soil added in two equal doses at 30 and 45 days after transplanting. All other agronomic practices were applied as recommended for rice under saline soil during the growing season.

### 2.2. Measurements

At the heading stage, plants of five hills were randomly taken from each plot to estimate the following characters:

1. Root characteristics: At heading, plant samples involving root were carefully taken to determine root length (cm), root volume (cm^3^), and root thickness (mm). Root length was determined as the length of the root from the base of the plant to the tip of the main axis of primary root. Volume of the root system per plant was determined in cubic centimeters using a standard column. To measure root thickness, the average diameter (mm) of the tip portion (about 1 cm from the tip) of three random secondary roots at the middle position of the plant root was estimated.
2. Growth and physiological characters: Leaf relative water content (RWC %) was calculated based on the described methods by Yamasaki and Dillenburg [41].

(1) $$\mathrm{RWC}\%=\frac{\mathrm{FW}-\mathrm{DW}}{\mathrm{TW}-\mathrm{DW}}\times 100$$

where FW = fresh weight of leaf; DW = dry weight; TW = turgor weight.

Turgor weight was measured in leaf discs that were floated in distilled water for 6 h in Petri dishes under laboratory light and temperature conditions. Then, leaf discs were blotted before weighing, and finally placed in an oven at 105 °C for 48 h to obtain the dry weight.

Chlorophyll content (Chl.) was measured using a SPAD meter (SPAD 502, Minolta, Japan). Leaf area index, plant height (cm), and dry matter production hill^−1^ were also measured at the same stage.

3. Ion selectivity: At heading, leaf samples were taken into the laboratory to analyze the Na^+^ and K^+^ contents as mg g^−1^ dry weight as well as to measure the Na^+^/K^+^ ratio according to Jackson [42].
4. Yield and its components: At harvest, number of panicles hill^−1^, panicle length (cm), number of unfilled grains panicle^−1^, number of filled grains panicle^−1^, 1000-grain weight (g), and grain weight/ panicle (g) were estimated from each plot. For grain yield, six inner rows from each plot were harvested, dried, threshed, and the grain, straw, and biological yields were determined. Then, yields/ha were calculated at 14% moisture content. Harvest index % was estimated as follows:

(2) $$\mathrm{Harvest}\;\mathrm{index}\;\%\;=\frac{\mathrm{Grain}\;\mathrm{yield}\;\mathrm{per}\;\mathrm{ha}}{\mathrm{Biological}\;\mathrm{yield}\;\mathrm{per}\;\mathrm{ha}}\times 100$$

### 2.3. Statistical Analysis

The obtained data were subjected to analysis of variance according to Gomes and Gomes [43]. Treatment means were compared by Duncan’s multiple range test [44]. All statistical analysis was performed using the analysis of variance technique by means of the “COSTAT” computer software package.

## 3. Results

### 3.1. Root Characters

Root characters of rice varieties showed a highly significant difference with respect to salinity stress conditions during the two growing seasons of the study. Data in Table 2 showed that root thickness, root volume, and root length were highly significantly affected by the application of both nano-silicon (NPs-Si) and nano-selenium nanoparticles (NPs-Se) during the two seasons while Giza 178 was superior to Giza 177 for the three root characteristics. On the other hand, soaking grains at NPs-Se treatment recorded the highest results for the three root characteristics compared to other application treatments during both seasons, followed by NPs-Si foliar at BT and NPs-Se foliar at BT for root thickness in 2018 and 2019 seasons and also for root length in 2018. Control treatment had the lowest results of the three root characteristics in both seasons. The interaction effects were highly significant for root thickness and root volume, while were not significant for root length in the two seasons.

Regarding the interaction effect of NPs-Si and NPs-Se treatments on root thickness and root volume of both rice varieties, soaking grains in NPs-Se resulted in the highest value of root thickness for Giza 178 (0.90 and 0.95 mm) and root volume (153.30 and 154.30 cm^3^) in 2018 and 2019, respectively (Table 3). This was followed by Giza 177 recorded of 0.83 and 0.81 mm for root thickness and 143.30 and 141.30 cm^3^ for root volume in both seasons, respectively. The foliar applications of NPs-Si and NPs-Se at the three growth stages (mid tillering stage MT; panicle initiation PI; mid booting stage BT) showed no significant differences in root thickness for both cultivars in 2018 with a slight difference in the 2019 season. On the other hand, the foliar application of NPs-Si and NPs-Se at BT surpassed the other stages (i.e., PI and MT) in root volume for Giza 178 during both seasons.

### 3.2. Growth and Physiological Traits

Data in Table 4 revealed that application of NPs-Si and NPs-Se had a highly significant effect on relative water content (RWC), leaf area index (LAI), and chlorophyll content for the two rice varieties grown in saline soil conditions during the 2018 and 2019 seasons. It was clear that Giza 178 was superior to Giza 177 for the three traits. Soaking grains in NPs-Si and NPs-Se, and the foliar application of NPs-Se at BT resulted in the highest relative water content in both seasons, while foliar application of NPs-Si at BT resulted in the highest leaf area index during 2018 and 2019, respectively. In addition, the highest values of chlorophyll content (SPAD value) were obtained by soaking the grains in NPs-Se compared to the other treatments.

**Figure 1:**
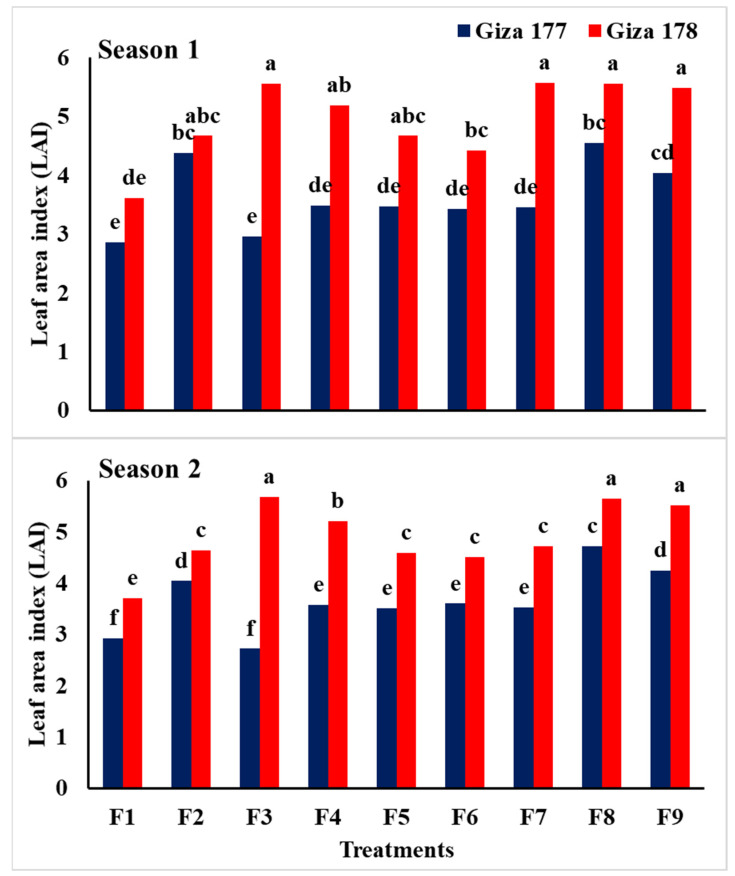
Interaction effect of NPs-Si and NPs-Se treatments on leaf area index (LAI) of the two rice varieties under saline soil conditions during the first and second seasons. F1 = control; F2 = soaking-NPs-Si; F3 = soaking-NPs-Se; F4 = foliar-NPs-Si at MT; F5 = foliar-NPs-Se at MT; F6 = foliar-NPs-Si at PI; F7 = foliar-NPs-Se at PI; F8 = foliar-NPs-Si at BT; F9 = foliar-NPs-Se at BT; NPs-Si = nanoparticles of silicon; NPs-Se = nanoparticles of selenium, MT = mid tillering stage; PI = panicle initiation; BT = mid booting stage. Means followed by the same letter were not significantly different for probability (*p*) ≤ 0.05.

**Figure 2:**
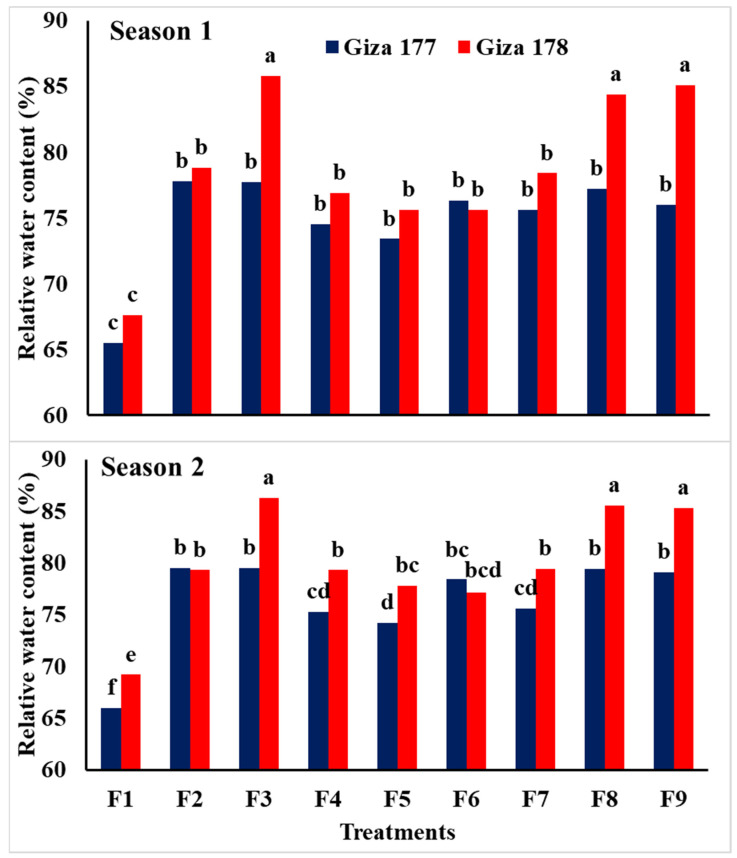
Interaction effect of NPs-Si and NPs-Se treatments on relative water content (%) of the two rice varieties under saline soil conditions during the first and second seasons. F1 = control; F2 = soaking-NPs-Si; F3 = soaking-NPs-Se; F4 = foliar-NPs-Si at MT; F5 = foliar-NPs-Se at MT; F6 = foliar-NPs-Si at PI; F7 = foliar-NPs-Se at PI; F8 = foliar-NPs-Si at BT; F9 = foliar-NPs-Se at BT; NPs-Si = nanoparticles of silicon; NPs-Se = nanoparticles of selenium, MT = mid tillering stage; PI = panicle initiation; BT = mid booting stage. Means followed by the same letter were not significantly different for probability (*p*) ≤ 0.05.

The interaction effects between the treatments (NPs-Si and NPs-Se) and varieties were highly significant for both relative water content and leaf area index, while it was not significant for chlorophyll content during the two seasons (Table 4, Figure 1 and Figure 2). The data indicated that soaking grains in NPs-Se and foliar application of NPs-Si at BT resulted in the highest LAI and RWC for plants of the Giza 178 variety in both seasons. In general, data showed that Giza 178 was superior to Giza 177 grown under different treatments, while the applications of NPs-Si and NPs-Se surpassed the control treatment for both the LAI and RWC of rice plants grown in saline soil.

Dry matter (g hill^−1^) and plant height (cm) of rice varieties showed highly significant differences with respect to salinity stress conditions during the two growing seasons of the study (Table 5). It was obviously clear that the Giza 178 variety had the same behavior of all the studied traits under salinity conditions, since it was superior to Giza 177 in both seasons. On the other hand, dry matter and plant height traits were significantly affected by NPs-Si and NPs-Se treatments during both seasons. Using the soaking method of the grains in NPs-Se and the foliar application of NPs-Si and NPs-Se at BI recorded the highest value for dry matter in both seasons, while foliar application of NPs-Si at BT and NPs-Se at PI resulted in the tallest plants during the 2018 and 2019 seasons. However, untreated plants with NPs-Si and NPs-Se recorded the lowest dry matter and shortest plant height during the two seasons. Concerning the interaction effect, no significant interaction effect was found for these two traits.

### 3.3. Yield and Yield Components

Yield and yield component traits of rice varieties showed highly significant differences with respect to the salinity stress during the two growing seasons in the study (Table 6). Number of panicles of the two rice varieties showed highly significant differences during the two growing seasons. It was clear that Giza 178 had the highest number of panicles in both seasons, and the tallest panicle during the 2019 season under salinity stress compared to Giza 177. However, Giza 177 was superior to Giza 178 in terms of panicle weight during the 2018 season.

Number of panicles and panicle weight were significantly affected by NPs-Si and NPs-Se treatments during both seasons (Table 6). The application of soaking grains in NPs-Se resulted in the highest number of panicle and panicle weight in both seasons. The same results were recorded by the foliar application of NPs-Si at BT, and foliar application of NPs-Se at PI for the highest number of panicles during 2018, and foliar application of NPs-Se at BT for the highest panicle weight during both seasons compared to other treatments. The effects of NPs-Si and NPs-Se were not significant on the panicle length compared to the control treatment. The interaction effect was not significant for these traits.

Data arranged in Table 7 and Table 8 revealed that the two tested rice varieties significantly differed in their number of filled grains, number of unfilled grains panicle^−1^, and 1000 grain weight in both seasons. Giza 178, as the salt tolerant rice variety, significantly exceeded Giza 177 (the salt sensitive variety) in the main yield component such as filled grains panicle^−1^, but Giza 177 significantly surpassed Giza 178 in the 1000-grain weight and number of unfilled grains panicle^−1^.

**Figure 3:**
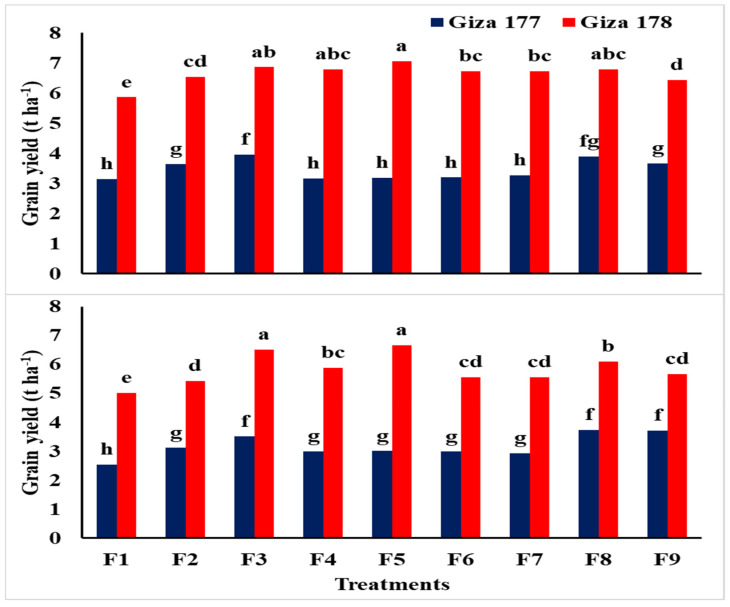
Interaction effects of NPs-Si and NPs-Se treatments on grain yield (t ha^−1^) of the two rice varieties in saline soil conditions during the first and second seasons. F1 = control; F2 = soaking-NPs-Si; F3 = soaking-NPs-Se; F4 = foliar-NPs-Si at MT; F5 = foliar-NPs-Se at MT; F6 = foliar-NPs-Si at PI; F7 = foliar-NPs-Se at PI; F8 = foliar-NPs-Si at BT; F9 = foliar-NPs-Se at BT; NPs-Si = nanoparticles of silicon; NPs-Se = nanoparticles of selenium, MT= mid tillering stage; PI = panicle initiation; BT = mid booting stage. Means followed by the same letter were not significantly different for probability (*p*) ≤ 0.05.

Data in Table 9 revealed that NPs-Si and NPs-Se treatments had a highly significant effect on grain yield ha^−1^, biological yield ha^−1^, and harvest index for both rice varieties under saline soil conditions in the 2018 and 2019 seasons. It was clear that Giza 178 was superior to Giza 177 for the yields and harvest index in both seasons of the study. On the other hand, grain yield ha^−1^, biological yield ha^−1^, and harvest index were significantly affected by NPs-Si and NPs-Se treatments during both seasons. For instance, grain soaking with NPs-Se and the foliar application of NPs-Si at BT recorded the highest grain yield of 5.41 and 5.34 t ha^−1^ in 2018 and 5.00 and 4.91 t ha^−1^ in 2019, respectively. This was followed by the foliar application of NPs-Se at MT and BI with no significant difference in both seasons. Additionally, the biological yield recorded the same trend as grain yield in both seasons of the study with a slight difference, since foliar application of NPs-Se at MT resulted in the highest value in 2019. Other NPs-Si and NPs-Se treatments recorded moderate results for grain and biological yields ha^−1^ as well as harvest index with no significant differences in both seasons of study. However, control treatment (without NPs) recorded the lowest values of these studied traits. The interaction effect was highly significant for grain yield, while it was not significant for both biological yield and harvest index in the two seasons (Figure 3).

Regarding the interaction effect of NPs-Si and NPs-Se treatments on grain yield t ha^−1^ of the two rice varieties under salinity conditions during the 2018 and 2019 seasons, data indicated that soaking grains with NPs-Se, and the foliar application of NPs-Se and NPs-Si at MT resulted in the highest grain yield t ha^−1^ for the Giza 178 variety in both seasons of the study (Figure 3). Meanwhile, the Giza 177 variety recorded the highest grain yield t ha^−1^ when soaking grains with NPs-Se, and the foliar application of NPs-Si and NPs-Se at BI showed no significant differences.

### 3.4. Ion Selectivity

The two studied rice varieties apparently varied in its Na^+^ and K^+^ leaf contents as well as Na^+^/K^+^ ratio (Table 10). Giza 177, as the salt sensitive variety, had the highest values of Na^+^ leaf content and Na^+^/K^+^ ratio, while it had the lowest values of K^+^ leaf content in both seasons. On the other hand, Giza 178, as the salt tolerant variety, contained the lowest Na^+^ leaf content combined with the lowest Na^+^/K^+^ ratio, while it had the highest K^+^ leaf content at the heading stage during both seasons under salt stress (Table 10).

On the other hand, it was noted that the ion selectivity adjusted by Na^+^ and K^+^ leaf contents as well as Na^+^/K^+^ ratio was positively affected by the different treatments of NPs-Si and NPs-Se (Table 10). Both NPs-Si and NPs-Se treatments, either foliar application or grain soaking, had positive effects on ion selectivity by reducing the Na^+^ uptake and raising the K^+^ uptake, consequently reducing the Na^+^/K^+^ ratio. Furthermore, the foliar application of NPs-Si at the boating stage significantly increased K^+^ and significantly reduced the Na^+^ and Na^+^/K^+^ ratio in rice plants grown under salt stress during both seasons without significant differences with those treated with the foliar application of NPs-Se at the same stage. The treatment of grain soaking in both NPs-Si and NPs-Se ranked second after the treatment of foliar application for both of them at the boating stage concerning the adjustment of ion selectivity in terms of low Na^+^/K^+^ ratio combined with high K^+^ and low Na ^+^ in the leaves of rice plants. Applying NPs at mid tillering (MT) or panicle initiation (PI) as a foliar application possessed the third order. The current finding supports the efficiency of NPs-Si and NPs-Se applications at the early growth stage in terms of seed priming, or at late growth stage during the boating stage to optimize the ion selectivity. The control treatment exerted the highest values of Na^+^ leaf content and Na^+^/K^+^ ratio with the lowest value of K^+^ leaf content compared to the NPs-Si and NPs-Se treatments in both seasons. The interaction effects between rice varieties and NPs of Si and Se treatments were not significant on the Na^+^, K^+^ and Na^+^/K^+^ ratio, indicating the independence of each factor as well as the two tested varieties had the same response.

## 4. Discussion

Salinity is a harmful environmental issue that is considered as one of the main causes of reduction in agricultural crop productivity through the dysfunction that occurs in plant physiological and biochemical processes as well as the antioxidant defenses as a result of the excessive production of reactive oxygen species (ROS). In addition, it can cause the instabilities of cell membranes and lipid peroxidation as a result of the increased Na+ ions along with increased ROS [13,45]. Salinity can increase the production of ROS in cellular organelles such as chloroplasts, peroxisomes, and mitochondria, which can negatively affect different processes such as transpiration, photosynthesis, stomatal conductance, and growth [13,46]. Nanotechnology has recently found its way into agricultural systems. Applications of mineral nanoparticles of various shapes and sizes have a significant potential for improving the performance of crop productivity [47] and moderating the environmental stress [48].

### 4.1. Root Characters

The foliar or soaking application of NPs-Si and NPs-Se has a favorable effect on the root length, root volume, and root thickness compared to the control treatment for both cultivars, particularly Giza 177. The reduction in the root length of plants grown in saline soil might be due to the high inhibitory effect of NaCl salt to root growth compared to that of shoot growth [49]. Excessive accumulation of Na^+^, Cl^−^, and SO_4_^−2^ ions in plant roots reduces the osmotic potential and limits water intake and plant growth, resulting in plant mortality in some situations [50]. Excessive salts impair physiological and biochemical activities such as photosynthesis, lipid metabolism, protein synthesis, and plant development [51]. However, roots appeared to be more sensitive to salt than the shoots. This observation suggests that the capacity to produce roots was more severely affected than the shoot. It is also suggestive of the more inhibitory effect of NaCl salt to root growth. It might also be due to the energy partitioning of the root, and this may be used as an indicator of the salinity tolerance in determining the salinity threshold of a particular variety. Since roots are the first target tissue to be confronted with excess concentrations of pollutants, toxic symptoms seem to appear more in the roots than in the shoots.

### 4.2. Growth and Physiological Traits

Relative water content, leaf area index, and chlorophyll content were significantly affected by NPs-Si and NPs-Se treatments in the two seasons. In general, salinity caused a considerable reduction in dry matter; however, application of NPs-Si increased plant height and leaf area compared with the untreated control plants. Growth parameters were increased with the application of NPs-Si and NPs-Se either under the normal condition or salinity stress condition (Table 3 and Table 4, Figure 1 and Figure 2). This can be due to the mitigation of adverse effects of salinity stress when NPs-Si and NPs-Se were applied [20]. In addition, the application of SiO_2_-NPs was observed to enhance leaf dry weight as well as chlorophyll, proline, and antioxidant levels of plants grown under salinity stress [52].

In this regard, Gong et al. [16,53] reported that dry matter yield of wheat plants was improved when Si was applied. According to Ma and Yamaji [54], the positive effect of Si is due to its deposition in the roots, decreases, and provides binding sites for metals, resulting in a reduction in the uptake and translocation of toxic metals and salts from the roots to the shoots. In an earlier study, increased K^+^ uptake and decreased Na^+^ uptake by the addition of Si in bean plants were the major mechanisms responsible for better growth of plants under salinity [55]. The physiological responses of rice plants to salinity stress at various developmental stages are therefore critical for identifying salinity tolerance in the cultivars. Low concentrations of Si and NPs-Si were more effective on plant growth at all salinity levels than high doses. These results are in context with Parveen and Ashraf [56], who found that the exogenously applied Si significantly enhanced plant water use efficiency (WUE), and slightly increased the photosynthetic rate under the saline stress condition in wheat and maize plants. This is likely due to the role of silicon in the enzyme activities and biochemical processes in plant tissues [57].

### 4.3. Yield and Yield Components

It was observed that the addition of NPs-Si or NPs-Se treatments either as soaking or foliar application showed a significant improvement in yield attributes of rice as a result of relieving the salinity hazard. Interestingly, foliar application of NPs-Si at the booting stage was more efficient to enhance yield components such as panicle weight, 1000-grain weight, and number of filled grains panicle^−1^ than foliar application of NPs-Se at the same growth stage. However, NPs-Se application such as grain soaking ranked third, and it was efficient to improve yield components more than the same treatment by NPs-Si. The high efficiency of NPs-Se such as grain soaking might be due to its affinity to enable plants from the first stage of salinity hazard in terms of a high osmotic pressure, reduce sodium uptake, increase K^+^ uptake in the terms of ion selectivity, and produce high seedling vigor with a free radical defense system. Since, the most affected yield component under salt stress for rice crop is grain filling [58], any variety that has the ability to reduce its panicle sterility under such a condition is considered as a salt tolerant variety. Moreover, approaches that have the ability to reduce panicle sterility such as foliar application of different nutrients in different modes or antioxidant plant hormones or polyamines of saccharides are recommended to be applied at the sensitive growth stage of plants. The beneficial effects of NPs-Si as a foliar application at the boating growth stage might be mainly attributed to reducing Na^+^ uptake, matching with increasing K^+^ and Ca^+2^ uptake, keeping healthy flag leaf, increasing photosynthesis with high net assimilation rate, and translocation from sources to sin. In this regard, Si is reported to enhance growth and yield of higher plants, particularly under biotic and abiotic stresses [16].

All of the above-mentioned benefits developed by both NPs-Si and NPs-Se as foliar application at the booting stage to ensure proper filling, and delaying early aging happened by salt stress by reducing free radical level in plans, which exhibited high panicle fertility with low sterility combined with heavy panicle resulted in high yield under salt stress. Exogenous application of Si and Se can modulate the unfavorable effects of environmental stresses on the yield of crop species [59]. Se can play a critical role in different plants, ultimately affecting plant yield, factors such as starch accumulation in chloroplast, resistance enhancement to oxidative stress, delaying of senescence, and water status adjustment under stress conditions as well as increase the antioxidative capacity [60]. It seems that the mechanical strength of plant tissues due to Si was because of the amorphous solid Si deposition on the cell wall layers [61]. The reduction in yield attributes may be attributed to the inhibiting effects of salinity on some metabolic processes in plant tissues [46,62]. Similarly, maize plants cultivated under salt stress yielded higher biological yields with the application of SiO_2_-NPs than plants grown without SiO_2_-NPs [63].

In the current study, the reduction in 1000 grain weight under salinity stress compared to the NP treatments was attributed to the inhibition in the uptake and transfer of the nutrition materials during the growth of grains and their filling periods. Moreover, salinity may cause severe damage to the ovary, and consequently can cause a reduction in the yield [64]. The application of Si and NPs-Si resulted in an increase in the yield of salt treated or untreated faba bean plants, as reported by [65,66]. The role of Si as a beneficial mineral nutrient for the reproductive growth of plants is well documented [67]. Generally, increased K uptake and decreased Na uptake by Si treatments was reported to be the major mechanisms responsible for better growth and yield of plants under salinity [68]. It has been observed that the supplemental Si improves the yield and reduces the plant biotic and abiotic stresses [69]. Some studies have shown that Si is an effective to mitigate the stress of salinity in different plant species such as cucumber [70], maize [71], tomato [72], and wheat [73]. Some possible mechanisms through which Si may increase salinity tolerance in plants include immobilization of toxic Na^+^ ions [74], reduced Na^+^ uptake in plants, and enhanced K^+^ uptake [18] as well as may result in a higher K^+^ and Na^+^ selectivity [75].

The harvest index (HI) was significantly affected by salinity stress. In this regard, HI values were decreased with increasing salinity. The present study showed that NPs-Si and NPs-Se treatments were the most effective in reducing the harmful effect of salinity on harvest index. In this regard, Kobraee et al. [76] stated that abiotic stress decreased harvest index. However, application of Si, on the other hand, caused an improvement in the harvest index of different crops under salinity stress [77]. Moreover, Kardoni et al. [65] and Parande et al. [78] found that application of Si improved the yield, yield components, and harvest index of faba bean under salt stress conditions.

It is clear from the results of all the studied traits that Giza 177, which is sensitive to salinity, gave superior results and was very close to Giza 178, which tolerated salinity, under the influence of NPs-Si and NPs-Se treatments compared to the control (without NPs). It is worth mentioning that applying the studied NPs of Si and Se positively improved the yield component of the salt sensitive Giza 177 rice variety as a result of improving its ion selectivity system, free radical defense system, and subsequently, high current photosynthesis developed during grain filling from healthy flag leaf. As has been cited, both Se and Si almost had the same mode of action to mitigate the salinity harmful on the plant and enhance plant growth, photosynthesis, and dry matter production; and subsequently can increase the yield under such condition by reducing the Na^+^ uptake and Cl^−^ deposition and improved K^+^ accumulation, releasing more antioxidant substances and improving metabolism and physiological performance [20,32].

### 4.4. Ion Selectivity

Regarding the ion selectivity response, rice varieties have the ability to decline some of the cation uptake such as Na^+^ as well as uptake other beneficial cations such as K^+^ due to its high affinity to the plant growth under salt stress conditions. The latter concept is ion selectivity, since the variety had low Na^+^/K^+^ ratio such as in Giza 178 (i.e., salt tolerant variety). A variety such as Giza 178 might have specific root ion transporters and anti- porters that could enable it to uptake more K^+^ and hold Na^+^ ions away from the root, which is well-known as the first defense against salts. Additionally, any rice variety combined with high ion selectivity can grow healthy under salt stress by avoiding ion toxicity, which can happen under such condition. The mechanisms for alleviating Na^+^ toxicity in leaves include limiting the absorption of Na^+^ from the soil, lessening the transport of Na^+^ into the xylem, storing Na^+^ in the lower part of the plant leaves such as sheath in cereal crops, isolating Na^+^ into the vacuole, and cycling Na^+^ from the plant shoots to roots [79].

The negative effect of salinity affects plants during two stages, the first is due to the high osmotic pressure and the second is ion toxicity due to more accumulation of some ions such as Na^+^ and Cl^−^. As seen in the current investigation, both NPs-Si and NPs-Se showed a great benefit in improving the ion selectivity by reducing Na^+^ and increasing K^+^ uptake, particularly when applied as a foliar application at late growth stage during the boating stage. Additionally, grain soaking by both NPs-Si and NPs-Se as early treatment was found to be efficient in improving the ion selectivity due to the improving seedling vigor, fast cell elongation and growth, and osmotic protection, which enables the plants with the developed transporter and Antiporter associated with the positive ion selectivity on the cell membrane and vacuole wall. Furthermore, the beneficial effects of both NPs-Si and NPs-Se in reducing some hazard cations such Na and Cd in rice were due to the antagonism concept [32]. The application of SiO_2_-NPs reduced the Na^+^ ion toxicity and boosted plant development under salinity stress compared to plants that did not receive SiO_2_-NPs [80]. Applying Si increased the sclerenchyma cell formation and size in the root to enable plants to reduce the uptake of harmful ions such as Cd and Na^+^, which also explained the role of applying Si and Se, particularly as an early application by grain soaking for both of them [81]. Salt-affected soils have a low soil osmotic potential, causing nutritional imbalance in plants and increasing specific ionic toxicity. In this context, the usage of NPs could be a beneficial approach to address the growing problem of soil salinity.

## 5. Conclusions

In conclusion, the results highlight the role of NPs-Si and NPs-Se in regulating salinity responses, and indicate that the application of NPs-Si and NPs-Se could protect rice plants against the hazardous effect of salinity. There were no meaningful differences between the NPs-Si and NPs-Se applications; both forms were effective compared to the control in most measured characteristics.

Soaking and foliar applications of NPs-Si and NPs-Se are an efficient option to improve saline tolerance and rice crop yield. Favorable effects of Se/Si-NPs on root improvement, growth efficiency, and yield parameters at different growth stages have been attributed to (1) protection of pigments to increase photosynthetic capacity; (2) accumulation of assimilates to protect osmosis; (3) activation of an anti-oxidative system to eliminate reactive oxygen species ROS; (4) enhancing water use efficiency level for improvement of root biomass and maintenance of proper osmotic status of the cells; and (5) accumulation of biochemical compounds (total phenolic, anthocyanin contents, and antioxidant capacity). However, many new studies are required for the identification of the efficiency of NPs-Si and NPs-Se, especially in saline soils before practical programs can be realized on a large scale.

## Figures and Tables

**Table 1:** Chemical analysis of the experimental soil during the seasons of 2018 and 2019.

	Parameters	pH	EC (dS m^−1^)	OM (%)	Soluble Cations (meq L^−1^)				Soluble Anions (meq L^−1^)		
Seasons					Ca^++^	Mg^++^	K^+^	Na^+^	HCO_3_^−^	Cl^−^	SO_4_^2−^
**2018**		8.20	7.20	162	10.8	8.4	0.50	64	9.34	68.5	6.33
**2019**		8.26	7.00	1.60	6.4	6.2	0.70	66	8.64	64.6	5.33

**Table 2:** Effect of NPs-Si and NPs-Se treatments on root thickness, root volume, and root length of two rice varieties under saline soil conditions in the 2018 (S1) and 2019 (S2) seasons.

	Traits	Root Thickness (mm)		Root Volume (cm^3^)		Root Length (cm)	
Seasons		S1	S2	S1	S2	S1	S2
**Rice Varieties**							
**Giza 177**		0.747 ^b^	0.763 ^b^	92.02 ^b^	93.5 ^b^	18.1 ^b^	19.0 ^b^
**Giza 178**		0.798 ^a^	0.810 ^a^	130.0 ^a^	129.6 ^a^	25.5 ^a^	26.2 ^a^
**F test**		**	**	**	**	**	**
**NPs-Si and NPs-Se Treatments**							
**Control**		0.426 ^c^	0.486 ^e^	74.9 ^f^	75.8 ^f^	16.6 ^c^	17.5 ^c^
**Soaking-NPs-Si**		0.78 ^b^	0.821 ^b,c^	113.3 ^c,d^	107.6 ^d^	22.1 ^b^	22.5 ^b^
**Soaking-NPs-Se**		0.866 ^a^	0.923 ^a^	148.3 ^a^	147.8 ^a^	26.1 ^a^	27.3 ^a^
**Foliar-NPs-Si at MT**		0.778 ^b^	0.740 ^d^	93.30 ^e^	94.80 ^e^	20.5 ^b^	21.3 ^b^
**Foliar-NPs-Se at MT**		0.781 ^b^	0.743 ^d^	104.1 ^d^	106.8 ^d^	20.8 ^b^	21.6 ^b^
**Foliar-NPs-Si at PI**		0.843 ^a,b^	0.793 ^c,d^	105.0 ^d^	106.1 ^d^	21.3 ^b^	22.0 ^b^
**Foliar-NPs-Se at PI**		0.793 ^b^	0.855 ^b,c^	111.6 ^c,d^	128.1 ^b^	21.8 ^b^	22.6 ^b^
**Foliar-NPs-Si at BT**		0.846 ^a,b^	0.876 ^a,b^	130.0 ^b^	119.1 ^c^	23.8 ^a,b^	24.6 ^b^
**Foliar-NPs-Se at BT**		0.84 ^a,b^	0.845 ^b,c^	118.3 ^c^	117.6 ^c^	23.5 ^a,b^	24.1 ^b^
**F test**		**	**	**	**	**	**
**Interaction**		**	**	**	**	ns	ns

NPs-Si = nanoparticles of silicon; NPs-Se = nanoparticles of selenium, MT= mid tillering stage; PI = panicle initiation; BT = mid booting stage. Means followed by the same letter in each parameter were not significantly different for probability (*p*) ≤ 0.05. *p* ≥ 0.05 = ns; ** = *p* ≤ 0.01.

**Table 3:** Interaction effects of NPs-Si and NPs-Se treatments on root thickness and root volume of the two rice varieties under saline soil conditions.

	Traits	Root Thickness (mm)		Root Volume (cm^3^)	
Treatments		Giza 177	Giza 178	Giza 177	Giza 178
**Season 2018**					
**Control**		0.29 ^d^	0.56 ^c^	56.60 ^g^	93.30 ^f^
**Soaking-NPs-Si**		0.79 ^a,b^	0.76 ^b^	86.60 ^f^	140.00 ^a,b^
**Soaking-NPs-Se**		0.83 ^a,b^	0.90 ^a^	143.30 ^a,b^	153.30 ^a^
**Foliar-NPs-Si at MT**		0.75 ^b^	0.80 ^a,b^	80.10 ^f^	106.60 ^e^
**Foliar-NPs-Se at MT**		0.75 ^b^	0.81 ^a,b^	81.60 ^f^	126.60 ^c,d^
**Foliar-NPs-Si at PI**		0.84 ^a,b^	0.84 ^a,b^	83.30 ^f^	126.60 ^c,d^
**Foliar-NPs-Se at PI**		0.79 ^a,b^	0.79 ^a,b^	86.60 ^f^	136.60 ^b,c^
**Foliar-NPs-Si at BT**		0.84 ^a,b^	0.85 ^a,b^	116.60 ^d^	143.30 ^a,b^
**Foliar-NPs-Se at BT**		0.83 ^a,b^	0.85 ^a,b^	93.30 ^f^	143.30 ^a,b^
**Season 2019**					
**Control**		0.336 ^f^	0.636 ^e^	57.30 ^j^	94.30 ^g^
**Soaking-NPs-Si**		0.82 ^b,c,d^	0.82 ^b,c,d^	87.30 ^h^	128.00 ^d^
**Soaking-NPs-Se**		0.89 ^a,b^	0.95 ^a,b^	141.30 ^b,c^	154.30 ^a^
**Foliar- NPs-Si at MT**		0.76 ^c,d^	0.71 ^d^	81.60 ^i^	108.00 ^f^
**Foliar- NPs-Se at MT**		0.75 ^c,d^	0.73 ^c,d^	85.60 ^h,i^	128.00 ^d^
**Foliar- NPs-Si at PI**		0.79 ^b,c,d^	0.79 ^b,c,d^	85.00 ^h,i^	127.30 ^d^
**Foliar- NPs-Se at PI**		0.80 ^b,c,d^	0.90 ^a,b^	118.00 ^e^	138.30 ^c^
**Foliar- NPs-Si at BT**		0.85 ^a,b,c^	0.90 ^a,b^	94.00 ^g^	144.30 ^b^
**Foliar- NPs-Se at BT**		0.83 ^a,b,c^	0.84 ^a,b,c^	91.30 ^g^	144.00 ^b^

NPs-Si = nanoparticles of silicon; NPs-Se = nanoparticles of selenium, MT= mid tillering stage; PI = panicle initiation; BT = mid booting stage. Means followed by the same letter in each parameter were not significantly different for probability (*p*) ≤ 0.05.

**Table 4:** Effect of NPs-Si and NPs-Se treatments on relative water content, leaf area index, and chlorophyll content of two rice varieties under saline soil conditions in the 2018 (S1) and 2019 (S2) seasons.

	Traits	Relative Water Content (%)		Leaf Area Index (LAI)		Chlorophyll Content (SPAD)	
Seasons							
Seasons		S1	S2	S1	S2	S1	S2
**Rice varieties**							
**Giza 177**		74.9 ^b^	76.3 ^b^	3.62 ^b^	3.65 ^b^	41.16 ^b^	41.9 ^b^
**Giza 178**		78.7 ^a^	79.9 ^a^	4.86 ^a^	4.91 ^a^	42.77 ^a^	42.3 ^a^
**F test**		**	**	**	**	**	**
**NPs-Si and NPs-Se treatments**							
**Control**		66.5 ^c^	67.6 ^d^	3.23 ^d^	3.32 ^e^	38.6 ^c^	38.6 ^d^
**Soaking-NPs-Si**		78.3 ^a,b^	79.4 ^b^	4.52 ^b,c^	4.33 ^c^	42.2 ^b^	42.3 ^b,c^
**Soaking-NPs-Se**		81.7 ^a^	82.9 ^a^	4.26 ^b,c^	4.20 ^c,d^	44.1 ^a^	43.7 ^a^
**Foliar-NPs-Si at MT**		75.7 ^b^	77.3 ^b,c^	4.33 ^b,c^	4.39 ^c^	42.15 ^b^	42.5 ^a,b,c^
**Foliar-NPs-Se at MT**		74.5 ^b^	76.0 ^c^	4.07 ^c^	4.04 ^d^	41.9 ^b^	41.7 ^c^
**Foliar-NPs-Si at PI**		75.9 ^b^	77.7 ^b,c^	3.93 ^c^	4.06 ^d^	41.5 ^b^	42.2 ^b,c^
**Foliar-NPs-Se at PI**		77.0 ^b^	77.5 ^b,c^	4.01 ^c^	4.12 ^d^	41.6 ^b^	42.1 ^b,c^
**Foliar-NPs-Si at BT**		80.8 ^a^	82.4 ^a^	5.06 ^a^	5.18 ^a^	42.6 ^b^	43.4 ^a,b^
**Foliar-NPs-Se at BT**		80.6 ^a^	82.2 ^a^	4.76 ^a,b^	4.87 ^b^	42.7 ^b^	42.6 ^a,b,c^
**F test**		**	**	**	**	*	**
**Interaction**		**	**	**	**	ns	ns

NPs-Si = nanoparticles of silicon; NPs-Se = nanoparticles of selenium, MT= mid tillering stage; PI = panicle initiation; BT = mid booting stage. Means followed by the same letter in each parameter were not significantly different for probability (*p*) ≤ 0.05. *p* ≥ 0.05 = ns; * = *p* ≤ 0.05; ** = *p* ≤ 0.01.

**Table 5:** Effect of NPs-Si and NPs-Se treatments on dry matter and plant height of two rice varieties under saline soil conditions in the 2018 (S1) and 2019 (S2) seasons.

	Traits	Dry Matter (g Hill^−1^)		Plant Height (cm)	
Seasons		S1	S2	S1	S2
**Rice varieties**					
**Giza 177**		28.9 ^b^	30.3 ^b^	85.0 ^b^	86.0 ^b^
**Giza 178**		38.5 ^a^	39.7 ^a^	87.6 ^a^	88.1 ^a^
**F test**		**	**	**	**
**NPs-Si and NPs-Se treatments**					
**Control**		27.4 ^c^	27.6 ^d^	83.6 ^c^	84.4 ^c^
**Soaking-NPs-Si**		34.5 ^a,b^	35.3 ^b^	85.0 ^b,c^	85.7 ^b,c^
**Soaking-NPs-Se**		39.7 ^a^	40.6 ^a^	86.2 ^a,b^	86.9 ^a,b^
**Foliar-NPs-Si at MT**		33.1 ^a,b,c^	33.4 ^b,c^	86.6 ^a,b^	87.4 ^a,b^
**Foliar-NPs-Se at MT**		31.6 ^b,c^	33.7 ^b,c^	86.7 ^a,b^	87.4 ^a,b^
**Foliar-NPs-Si at PI**		31.1 ^b,c^	31.7 ^c^	86.6 ^a,b^	87.3 ^a,b^
**Foliar-NPs-Se at PI**		33.1 ^a,b,c^	35.2 ^b^	87.8 ^a^	88.5 ^a^
**Foliar-NPs-Si at BT**		37.3 ^a,b^	39.1 ^a^	87.9 ^a^	88.7 ^a^
**Foliar-NPs-Se at BT**		35.6 ^a,b^	38.3 ^a^	86.5 ^a,b^	87.2 ^a,b^
**F test**		**	**	**	**
**Interaction**		ns	ns	ns	ns

NPs-Si = nanoparticles of silicon; NPs-Se = nanoparticles of selenium, MT= mid tillering stage; PI = panicle initiation; BT = mid booting stage. Means followed by the same letter in each parameter were not significantly different for probability (*p*) ≤ 0.05. *p* ≥ 0.05 = ns; ** = *p* ≤ 0.01.

**Table 6:** Effect of NPs-Si and NPs-Se treatments on number of panicles, panicle weight, and panicle length of two rice varieties under saline soil conditions in the 2018 (S1) and 2019 (S2) seasons.

	Traits	Number of Panicles Hill^−1^		Panicle Weight		Panicle Length	
Seasons		S1	S2	S1	S2	S1	S2
**Rice varieties**							
**Giza 177**		16.37 ^b^	15.6 ^b^	2.80 ^a^	2.70	19.9 ^b^	18.4 ^b^
**Giza 178**		21.8 ^a^	20.4 ^a^	2.64 ^b^	2.47	21.3 ^a^	21.4 ^a^
**F test**		**	**	**	ns	**	**
**NPs-Si and NPs-Se treatments**							
**Control**		17.00 ^c^	16.2 ^c^	2.41 ^d^	2.17 ^c^	19.61	19.3
**Soaking-NPs-Si**		18.9 ^a,b^	18.06 ^b,c^	2.83 ^a,b,c^	2.54 ^b^	21.0	20.4
**Soaking-NPs-Se**		20.4 ^a^	20.7 ^a^	2.99 ^a^	2.91 ^a^	21.3	20.4
**Foliar-NPs-Si at MT**		18.5 ^a,b^	19.1 ^a,b^	2.69 ^b,c^	2.58 ^b^	20.6	19.8
**Foliar-NPs-Se at MT**		18.5 ^a,b^	17.9 ^b,c^	2.73 ^b,c^	2.47 ^b^	20.6	19.1
**Foliar-NPs-Si at PI**		18.6 ^a,b^	17.4 ^b,c^	2.50 ^d^	2.60 ^b^	20.0	20.4
**Foliar-NPs-Se at PI**		18.9 ^a,b^	18.3 ^b^	2.65 ^c^	2.56 ^b^	20.6	20.5
**Foliar-NPs-Si at BT**		20.5 ^a^	19.2 ^a,b^	2.86 ^a,b^	2.78 ^a^	21.1	19.7
**Foliar-NPs-Se at BT**		19.8 ^a^	17.8 ^b,c^	2.82 ^a,b,c^	2.80 ^a^	21.0	20.0
**F test**		**	**	**	**	ns	ns
**Interaction**		ns	ns	ns	ns	ns	ns

NPs-Si = nanoparticles of silicon; NPs-Se = nanoparticles of selenium, MT = mid tillering stage; PI = panicle initiation; BT = mid booting stage. Means followed by the same letter in each parameter were not significantly different for probability (*p*) ≤ 0.05. *p* ≥ 0.05 = ns; ** = *p* ≤ 0.01.

**Table 7:** Effect of NPs-Si and NPs-Se treatments on number of filled grains/panicle, number of unfilled grains/panicle, and 1000 grain weight of two rice varieties under saline soil conditions in the 2018 (S1) and 2019 (S2) seasons.

	Traits	Number of Filled Grains Panicle^−1^		Number of Unfilled Grains Panicle^−1^		1000-Grain Weight (g)	
Seasons							
Seasons		S1	S2	S1	S2	S1	S2
**Rice varieties**							
**Giza 177**		115.42 ^b^	93.01 ^b^	24.54 ^a^	23.0 ^a^	25.9 ^a^	25.7 ^a^
**Giza 178**		127.0 ^a^	116.1 ^a^	17.91 ^b^	15.2 ^b^	18.7 ^b^	19.23 ^b^
**F test**		**	**	**	**	**	**
**NPs-Si and NPs-Se treatments**							
**Control**		103.1 ^e^	90.9 ^d^	34.2 ^a^	32.8 ^a^	19.50 ^e^	19.40 ^c^
**Soaking-NPs-Si**		123.4 ^c^	100.0 ^c^	21.9 ^b^	20.8 ^b^	22.2 ^b,c,d^	22.30 ^b^
**Soaking-NPs-Se**		127.85 ^b^	108.0 ^b^	16.8 ^d^	16.6 ^c,d^	23.20 ^a^	23.50 ^a,b^
**Foliar-NPs-Si at MT**		118.6 ^d^	102.7 ^c^	21.8 ^b^	19.6 ^b,c^	22.0 ^c,d^	22.20 ^b^
**Foliar-NPs-Se at MT**		124.1 ^b^	104.8 ^b,c^	19.1 ^c^	20.9 ^b^	22.6 ^b,c^	22.40 ^b^
**Foliar-NPs-Si at PI**		116.0 ^d^	103.8 ^b,c^	21.4 ^b^	16.6 ^c,d^	22.2 ^b,c,d^	22.10 ^b^
**Foliar-NPs-Se at PI**		117.7 ^d^	103.9 ^b,c^	19.7 ^b,c^	17.2 ^b,c,d^	22.1 ^c,d^	22.30 ^b^
**Foliar-NPs-Si at BT**		133.3 ^a^	114.1 ^a^	15.8 ^d^	12.1 ^e^	23.9 ^a^	24.60 ^a^
**Foliar-NPs-Se at BT**		130.7 ^a,b^	113.6 ^a^	20.0 ^b,c^	15.2 ^d^	23.3 ^a^	23.80 ^a^
**F test**		**	**	**	**	**	*
**Interaction**		**	ns	**	**	ns	ns

NPs-Si = nanoparticles of silicon; NPs-Se = nanoparticles of selenium, MT= mid tillering stage; PI = panicle initiation; BT = mid booting stage. Means followed by the same letter in each parameter were not significantly different for probability (*p*) ≤ 0.05. *p* ≥ 0.05 = ns; * = *p* ≤ 0.05; ** = *p* ≤ 0.01.

**Table 8:** Effect of the interaction between NPs-Si, NPs-Se, and rice varieties on the number of filled and unfilled grains panicle^−1^ in the 2018 (S1) and 2019 (S2) seasons.

	Traits	Number of Filled Grains Panicle ^−1^		Number of Unfilled Grains Panicle^−1^			
Treatments		Giza 177	Giza 178	Giza 177	Giza 178	Giza 177	Giza 178
		S1		S1		S2	
**Control**		91.7 ^g^	114.5 ^f^	39.3 ^a^	29.1 ^b^	42.16 ^a^	23.4 ^b^
**Soaking-NPs-Si**		118.8 ^e,f^	127.9 ^b,c^	19.5 ^e,f,g^	24.1 ^c^	23.3 ^b,c^	18.4 ^b,c,d^
**Soaking-NPs-Se**		123.1 ^d,e^	132.4 ^ab^	16.35 ^g,h^	17.37 ^f,g,h^	18.7 ^b,c,d^	14.2 ^c,d^
**Foliar-NPs-Si at MT**		111.6 ^f^	125.5 ^b,c,d^	23.47 ^c,d^	20.03 ^d,e,f^	23.5 ^b^	15.7 ^c,d^
**Foliar-NPs-Se at MT**		111.6 ^f^	130.10 ^a,b^	25.67 ^c^	12.76 ^i^	20.9 ^b,c^	20.90 ^b,c^
**Foliar-NPs-Si at PI**		113.3 ^f^	118.7 ^e,f^	24.10 ^c^	18.9 ^e,f,g^	20.7 ^b,c^	12.4 ^d,e^
**Foliar-NPs-Se at PI**		113.2 ^f^	122.2 ^c,d,e^	20.84 ^d,e,f^	18.8 ^e,f,g^	18.5 ^b,c,d^	16.0 ^c,d^
**Foliar-NPs-Si at BT**		129.4 ^b^	136.6 ^a^	17.11 ^f,g,h^	14.30 ^h,i^	15.4 ^c,d^	8.76 ^e^
**Foliar-NPs-Se at BT**		126.1 ^b,c^	135.1 ^a^	18.46 ^f,g^	21.90 ^c,d,e^	15.4 ^c,d^	15.0 ^c,d^

NPs-Si = nanoparticles of silicon; NPs-Se = nanoparticles of selenium, MT= mid tillering stage; PI = panicle initiation; BT = mid booting stage. Means followed by the same letter in each parameter were not significantly different for probability (*p*) ≤ 0.05.

**Table 9:** Effect of NPs-Si and NPs-Se treatments on grain and biological yields, and harvest index of two rice varieties in saline soil during the 2018 (S1) and 2019 (S2) seasons.

	Traits	Grain Yield (t ha^−1^)		Biological Yield (t ha^−1^)		Harvest Index (%)	
Seasons		S1	S2	S1	S2	S1	S2
**Rice varieties**							
**Giza 177**		3.45 ^b^	3.16 ^b^	8.06 ^b^	10.1 ^b^	0.428 ^b^	0.310 ^b^
**Giza 178**		6.64 ^a^	5.81 ^a^	13.5 ^a^	12.6 ^a^	0.488 ^a^	0.456 ^a^
**F test**		**	**	**	**	**	**
**NPs-Si and NPs-Se treatments**							
**Control**		4.49 ^c^	3.78 ^d^	9.94 ^d^	9.95 ^d^	0.445 ^b^	0.370 ^b^
**Soaking-NPs-Si**		5.08 ^b^	4.26 ^c^	10.6 ^b,c^	10.7 ^c^	0.477 ^a^	0.388 ^a,b^
**Soaking-NPs-Se**		5.41 ^a^	5.00 ^a^	11.5 ^a^	11.7 ^a,b^	0.464 ^a,b^	0.416 ^a^
**Foliar-NPs-Si at MT**		4.97 ^b^	4.43 ^c^	10.4 ^c^	11.6 ^a,b^	0.464 ^a,b^	0.373 ^b^
**Foliar-NPs-Se at MT**		5.12 ^b^	4.83 ^a,b^	11.0 ^b^	11.9 ^a^	0.452 ^a,b^	0.390 ^a,b^
**Foliar-NPs-Si at PI**		4.96 ^b^	4.26 ^c^	10.6 ^b,c^	11.5 ^a,b^	0.455 ^a,b^	0.363 ^b^
**Foliar-NPs-Se at PI**		4.99 ^b^	4.24 ^c^	10.7 ^b,c^	11.2 ^b^	0.456 ^a,b^	0.370 ^b^
**Foliar-NPs-Si at BT**		5.34 ^a^	4.91 ^a^	11.4 ^a^	11.9 ^a^	0.460 ^a,b^	0.406 ^a^
**Foliar-NPs-Se at BT**		5.04 ^b^	4.68 ^b^	10.9 ^b^	11.6 ^a,b^	0.453 ^a,b^	0.398 ^a,b^
**F test**		**	**	**	**	**	**
**Interaction**		**	**	ns	ns	ns	ns

NPs-Si = nanoparticles of silicon; NPs-Se = nanoparticles of selenium, MT= mid tillering stage; PI = panicle initiation; BT = mid booting stage. Means followed by the same letter in each parameter were not significantly different for probability (*p*) ≤ 0.05. *p* ≥ 0.05 = ns; ** = *p* ≤ 0.01.

**Table 10:** Na^+^ and K^+^ leaf content (mg g dry weight) and Na^+^/K^+^ ratio as affected by rice varieties and treatments of NPs-Si and NPs-Se during the 2019 (S1) and 2020 (S2) seasons.

	Traits	Na		K^+^		Na^+^/K^+^ ratio	
Seasons		S1	S2	S1	S2	S1	S2
**Rice varieties**							
**Giza 177**		4.13 ^a^	4.32 ^a^	2.41 ^b^	2.42 ^b^	1.71 ^a^	1.79 ^a^
**Giza 178**		2.37 ^b^	2.14 ^b^	4.11 ^a^	4.38 ^a^	0.58 ^b^	0.49 ^b^
**F test**		**	**	**	**	**	**
**NPs-Si and NPs-Se treatments**							
**Control**		4.56 ^a^	4.33 ^a^	2.31 ^d^	2.42 ^d^	1.97 ^a^	1.79 ^a^
**Soaking-NPs-Si**		3.07 ^c^	3.00 ^c^	3.31 ^b^	3.70 ^b^	0.92 ^c^	0.85 ^c^
**Soaking-NPs-Se**		3.00 ^c^	3.05 ^c^	3.35 ^b^	3.62 ^b^	0.90 ^c^	0.84 ^c^
**Foliar-NPs-Si at MT**		3.56 ^b^	3.67 ^b^	3.15 ^b^	3.15 ^b^	1.13 ^b^	1.17 ^b^
**Foliar-NPs-Se at MT**		3.63 ^b^	3.72 ^b^	3.00 ^b^	3.21 ^b^	1.21 ^b^	1.16 ^b^
**Foliar-NPs-Si at PI**		3.34 ^b^	3.56 ^b^	3.14 ^b^	3.16 ^b^	1.06 ^b^	1.13 ^b^
**Foliar-NPs-Se at PI**		3.45 ^b^	3.52 ^b^	3.16 ^b^	3.07 ^b^	1.09 ^b^	1.15 ^b^
**Foliar-NPs-Si at BT**		2.15 ^d^	2.11 ^d^	4.36 ^a^	4.25 ^a^	0.49 ^d^	0.50 ^d^
**Foliar-NPs-Se at BT**		2.33 ^d^	2.25 ^d^	4.00 ^a^	4.10 ^a^	0.58 ^d^	0.55 ^d^
**F test**		**	**	**	**	**	**
**Interaction**		ns	ns	ns	ns	ns	ns

NPs-Si = nanoparticles of silicon; NPs-Se = nanoparticles of selenium, MT= mid tillering stage; PI = panicle initiation; BT = mid booting stage. Means followed by the same letter in each parameter were not significantly different for probability (*p*) ≤ 0.05. *p* ≥ 0.05 = ns; ** = *p* ≤ 0.01.

## Data Availability

All data is presented within the article.
